# Supplementary material for: Hepatocellular senescence induces multi-organ senescence and dysfunction via TGFβ
Source: Nat Cell Biol. 2024 Nov 13;26(12):2075–83. doi: 10.1038/s41556-024-01543-3 (PMC11628396; doi:10.1038/s41556-024-01543-3)
Supplement: Supplementary file 1 — Reporting Summary [file 41556_2024_1543_MOESM1_ESM.pdf]

nature portfolio

Corresponding author(s): Thomas G Bird
Last updated by author(s): 30/8/24

Reporting Summary

Nature Portfolio wishes to improve the reproducibility of the work that we publish. This form provides structure for consistency and transparency in reporting. For further information on Nature Portfolio policies, see our Editorial Policies and the Editorial Policy Checklist.

Statistics

For all statistical analyses, confirm that the following items are present in the figure legend, table legend, main text, or Methods section.

n/a	Confirmed	
☐	☒	The exact sample size (*n*) for each experimental group/condition, given as a discrete number and unit of measurement
☐	☒	A statement on whether measurements were taken from distinct samples or whether the same sample was measured repeatedly
☐	☒	The statistical test(s) used AND whether they are one- or two-sided<br>*Only common tests should be described solely by name; describe more complex techniques in the Methods section.*
☐	☒	A description of all covariates tested
☐	☒	A description of any assumptions or corrections, such as tests of normality and adjustment for multiple comparisons
☐	☒	A full description of the statistical parameters including central tendency (e.g. means) or other basic estimates (e.g. regression coefficient) AND variation (e.g. standard deviation) or associated estimates of uncertainty (e.g. confidence intervals)
☐	☒	For null hypothesis testing, the test statistic (e.g. *F*, *t*, *r*) with confidence intervals, effect sizes, degrees of freedom and *P* value noted<br>*Give P values as exact values whenever suitable.*
☒	☐	For Bayesian analysis, information on the choice of priors and Markov chain Monte Carlo settings
☒	☐	For hierarchical and complex designs, identification of the appropriate level for tests and full reporting of outcomes
☒	☐	Estimates of effect sizes (e.g. Cohen's *d*, Pearson's *r*), indicating how they were calculated

Our web collection on statistics for biologists contains articles on many of the points above.

Software and code

Policy information about availability of computer code

Data collection	Open source code used with R environment
Data analysis	CellRanger (v.4.0), DropletUtils (v.1.10), Scater package (v.1.18) and the R environment (v.4.0) were used to analyse the scRNA-seq data. For the bulk RNA-seq, the R environment (v.3.4) was used to analyse the data, utilizing packages from Bioconductor (vXXX) and Metacore (https://portal.genego.com/). Raw data quality checks/trimming used FastQC (v0.11.7), FastP and FastQ Screen (v0.12.0). The reads were aligned to the mouse genome and annotation (vGRCm38.92) using HiSat2 (v2.1.0183).Determination and statistical analysis of expression levels was done by a combination of HTSeq (v0.9.1184), the R environment version (v3.4), utilizing packages from Bioconductor and differential gene expression analysis based on the negative binomial distribution using the DESeq2 package (v1.18.1186). Pathway Analysis was preformed using MetaCore (v) from Clarivate Analytics (https://portal.genego.com/).

For manuscripts utilizing custom algorithms or software that are central to the research but not yet described in published literature, software must be made available to editors and reviewers. We strongly encourage code deposition in a community repository (e.g. GitHub). See the Nature Portfolio guidelines for submitting code & software for further information.

Data

Policy information about availability of data

All manuscripts must include a data availability statement. This statement should provide the following information, where applicable:

- Accession codes, unique identifiers, or web links for publicly available datasets
- A description of any restrictions on data availability
- For clinical datasets or third party data, please ensure that the statement adheres to our policy

The fastq files and processed data for the single-cell RNAseq analysis of mouse kidney cells and bulk tissue transcriptomics in liver and kidney can be found on the Gene Expression Omnibus (GEO) repository; accession numbers: GSE189726, GSE267196 and GSE262705. Source data have been provided in Source Data. All other data generated and/or analysed during the current study are available from the corresponding author on reasonable request.

Research involving human participants, their data, or biological material

Policy information about studies with human participants or human data. See also policy information about sex, gender (identity/presentation), and sexual orientation and race, ethnicity and racism.

Reporting on sex and gender	Here we report the gender of the 34 patients enrolled in the study as defined by their self identification as recorded in medical records.
Reporting on race, ethnicity, or other socially relevant groupings	No socially constructed or socially relevant categorisations are made.
Population characteristics	atient covariates are described in table 1. Patients were recruited between 2010 and 2019. The diagnosis of acute indeterminate hepatitis was made by a combination of clinical, biochemical and histopathological criteria. The genders of the patients were balanced, with 7/10 male/females in each group.
Recruitment	The study included 34 consecutive patients with severe acute indeterminate hepatitis who were admitted to a single hospital and underwent transjugular liver biopsy or liver transplantation.
Ethics oversight	The study was approved by the London-Hampstead Research Ethics Committee (07/Q0501/50) and was in accordance with the declaration of Helsinki.

Note that full information on the approval of the study protocol must also be provided in the manuscript.

Field-specific reporting

Please select the one below that is the best fit for your research. If you are not sure, read the appropriate sections before making your selection.

☒ Life sciences ☐ Behavioural & social sciences ☐ Ecological, evolutionary & environmental sciences

For a reference copy of the document with all sections, see nature.com/documents/nr-reporting-summary-flat.pdf

Life sciences study design

All studies must disclose on these points even when the disclosure is negative.

Sample size	For animal experiments power calculations were not routinely performed; however, throughout animal biological replicate numbers were chosen based upon a predicted magnitude of response (50% effect size typically) taking into account the variability observed in pilot and prior experiments using control mice. This included analysis of mice induced outside the 8-12 week age induction window (additional data from these mice is available upon request). For all experiments the number of biological replicates ≥ 3 mice per cohort, specific n numbers for biological replicates are reported in figure legends for each experiment.
Data exclusions	Aside from the quality control steps using the pre-established exclusion criteria described no data was excluded from the analyses.
Replication	In animal experiments the N number reflects biological replicates, mice came from different litters and thus were not always sampled/induced on the same day. Staining and analysis was performed for all IHC analyses. Consistency was observed between between separate experiments when staining and analyses were batched. Combined data is reported throughout. Data from mice induced outside the 8-12 week age induction window was also analysed but not included here. This was typically consistent with those reported here also (additional data for these available upon request). All repeated attempts at induction over time were successful and no animals were excluded from analyses. For qRT-PCR data, N reflects biological replicates (mice) but within each plate, three technical replicates were performed for each biological replicate. For the serum treatment of wt MEFs (Fig. 4b), each data point in the "control" and "ΔMdm2Hep" groups represent technical replicates which derive from 2 different biological replicates i.e. plasma samples (in each group, there are 2 technical replicates for each one of the 2 biological replicates; this is highlighted specifically in the legend). In the "no plasma" group in Fig. 3b each dot represents a technical replicate (all technical replicates for this group were performed on the same plate). For the plasma treatment of the neural stem cell derived neuronal cells (Fig. 4c), each dot represents the mean of 3 technical replicates from one biological replicate i.e. plasma samples. In the "no

plasma" and "H2O2" groups in Fig. 4c, each dot represents a technical replicate (all technical replicates for these groups were performed on the same plate).

Randomization	Mice were manually split between experimental groups within litters and group sizes balanced overall. Biases were controlled as mice were assigned experimental group based on mouse ID which was assigned randomly by independent animal unit staff. Single colonies of inbred animals were used throughout, thus minimising bias between litters. Batches of AAV were created from common quantified stock solutions. All cohorts used in this study were age 8-12 weeks old unless otherwise stated in methods and male, except for the mice of the KrasG12D mouse model which were both male and female.
Blinding	Throughout animal welfare checks experimenters we not blinded to experimental groups. Group allocation is described above. Technical staff administering therapy were blinded to experimental conditions of mice. The experimenters were not blinded as induced mice with the LSL-RFP reporter develop red skin at the time of sampling. All subsequent tissue handling and analysis was blinded and/or performed using standardised automated analyses where possible.

Reporting for specific materials, systems and methods

We require information from authors about some types of materials, experimental systems and methods used in many studies. Here, indicate whether each material, system or method listed is relevant to your study. If you are not sure if a list item applies to your research, read the appropriate section before selecting a response.

Materials & experimental systems

n/a	Involved in the study	
☐	☒	Antibodies
☐	☒	Eukaryotic cell lines
☒	☐	Palaeontology and archaeology
☐	☒	Animals and other organisms
☐	☒	Clinical data
☒	☐	Dual use research of concern
☒	☐	Plants

Methods

n/a	Involved in the study	
☒	☐	ChIP-seq
☒	☐	Flow cytometry
☒	☐	MRI-based neuroimaging

Antibodies

Antibodies used

Antibody details are also provided in Table S3.
Antibody (primary)/ Supplier /Catalogue No /Dilution /Application /Clone number (monoclonals)
BrdU BD Biosciences 347580 1:250 IHC/IF B44
Caspase 3 Cell Signaling 9661 1:500 IHC NA
p21 Abcam ab107099 1:150 IHC/IF HUGO291
p53 Leica NCL-L-p53CM5p 1:750 IHC NA
pSMAD2 Cell Signalling 3108S 1:1000 WB 138D4
pSMAD3 Abcam ab52903 1:1000 WB EP823Y
pSTAT3 Cell Signaling 9131 1:100 IHC NA
RFP Tebu-Bio 600-401-379 1:1000 IHC/IF NA
SMAD2 Cell Signalling 5339 1:1000 WB D43B4
SMAD3 Cell Signalling 9513S 1:1000 WB NA
β-actin Sigma-Aldrich A2228 1:2000 WB AC-74
Lrp2 Abcam ab76969 1:1000 IF NA
Calb1 Abcam ab229915 1:1000 IF EPR22698-236

Antibody (secondary) Supplier Catalogue No Dilution Application
Donkey anti-mouse 488 Life Technologies A-21202 1:200 IHC
Donkey anti-mouse 555 Life Technologies A-31570 1:200 IHC
Donkey anti-rabbit 647 Life Technologies A-31573 1:200 IHC
Goat anti mouse HRP Cell Signalling 7076 1:3000 WB
Goat anti rabbit HRP Cell Signalling 7074 1:3000 WB
Horse anti-rabbit biotinylated Vector Labs BP-1100 R.T.U IHC

Validation

RFP; 600-401-379 – Manufacturer website: Application suitable for: IF and IHC. Reacts with: Mouse. Validated In house on RFP negative mouse liver tissue
BrdU; 347580 - relevant publication using antibody for IHC in mouse tissue from FFPE sections, validated in house in mice that did not receive BrdU cell labelling agent (negative control)
Cleaved caspase 3; 9661 - Manufacturer website: Applications: WB, IP, IHC-P, IF-IC, F. Reacts with: human, Mouse, Rat, Monkey. Mouse monoclonal antibody.
Lipocalin-2; AF1857 SP - Manufacturer website: Detects mouse Lipocalin-2/NGAL in direct ELISAs and Western blots. Goat polyclonal antibody, reacts with mouse.
p21; ab107099 - Manufacturer website: Suitable for: WB, IHC-P. Rat monoclonal, reacts with mouse and human. Validated in house on kidney tissue (IHC) from a p21 KO mouse and on whole liver lysate (WB) of another p21 KO mouse.
p53; NCL-L-p53CM5p - Manufacturer website: Suitable for IHC-P (HIER), rabbit polyclonal, reacts with mouse and rat.
pSMAD2; 3108S - Manufacturer website: Suitable for WB. Reacts with human, mouse, rat and mink. Rabbit monoclonal.

pSMAD3; ab52903 - Manufacturers website: Suitable for: WB, ICC/IF, IHC-P, Dot blot. Reacts with: Mouse, Human. Rabbit monoclonal.
pSTAT3; 9131 - Manufacturer website: Suitable for: WB, IP, ChIP. Reacts with human, mouse, rat and mink. Rabbit monoclonal.
SMAD2; 5339 - Manufacturer website: Suitable for: WB, IP, IF, ChIP, Flow cytometry. Reacts with human, mouse, rat and mink. Rabbit monoclonal.
SMAD3; 9513S - Manufacturer website: Suitable for: WB, IP, IF. Reacts with human, mouse and rat. Rabbit monoclonal.
β-actin; A2228 - Manufacturer website: Application suitable for: as loading control for western blots. It is a mouse monoclonal antibody.
Lrp2; Ab76969 - Manufactuer website: Suitable for: IHC-P. Reacts with: Mouse, Rat, Human, Monkey. Rabbit polyclonal.
Calb1; Ab 229915 - Manufactuer website: Suitable for: IHC-Fr, IHC-P, WB, IP. Reacts with: Mouse, Rat. Rabbit monoclonal

Eukaryotic cell lines

Policy information about cell lines and Sex and Gender in Research

Cell line source(s)

The WT murine embryonic fibroblasts (MEFs) were derived from E13.5-14.5 wt C57Bl/6J mouse embryos (gender not specified) and the neuronal stem cell - derived neuronal cells were derived from male human fetal neural crest progenitor cells as referenced.

Authentication

None of the cell lines were authenticated

Mycoplasma contamination

The wt MEFs were confirmed to be free of mycoplasma contamination at passage 4 by a PCR-based assay. The iPSCs-derived neuronal cells were isolated and differentiated as described in (Madgwick A. at al., 2015) and were not tested for mycoplasma contamination.

Commonly misidentified lines
(See ICLAC register)

No commonly misidentifed cell lines were used in this study

Animals and other research organisms

Policy information about studies involving animals; ARRIVE guidelines recommended for reporting animal research, and Sex and Gender in Research

Laboratory animals

The animals used in this study were on a mixed (129P2/OlaHsdWtsi;C57Bl/6J) background. Only male animals were used except for the experiments with the KrasG12D mouse model were both males and females were used. All mice used in this study were induced between 8-12 weeks of age. The mice used in this study carried the following transgenes: Mdm2tm2.1Glo, Gt(ROSA)26Sortm14(CAG-tdTomato)Hze and Krastm4Ty. All transgenic mice used were born in house.

Wild animals

This study did not involve wild animals.

Reporting on sex

Sex of animals is reported throughout. As outlined above only male animals were used except for the experiments with the KrasG12D mouse model were both males and females were used.

Field-collected samples

This study did not involve samples collected from the field.

Ethics oversight

All animal studies were performed in accordance with a UK Home Office project licence (70/8891 (protocol number 2) or PP0604995 (protocol number 3)) and were subject to review by the animal welfare and ethical review board of the University of Glasgow.

Note that full information on the approval of the study protocol must also be provided in the manuscript.

Clinical data

Policy information about clinical studies
All manuscripts should comply with the ICMJE guidelines for publication of clinical research and a completed CONSORT checklist must be included with all submissions.

Clinical trial registration

Details of a clinical study (non CTIMP) are reported. Clinical Trial Registration: the ethics pre-dates requirement for clinical trials registration (2007).

Study protocol

Entitled "Biomarkers of hepatic inflammation, senescence, and regeneration in severe acute hepatitis" is available upon request from Professor Jalan., Royal Free Hospital London

Data collection

Data collection was performed from patient notes both prospectively and retrospectively. Data collection form is available upon request from Professor Jalan, Royal Free Hospital London.

Outcomes

The study is observational upto 90 days post admission to hospital and patients continue to be followed up without prespecified endpoints to 90 days or until mortality.

Plants

Seed stocks

Report on the source of all seed stocks or other plant material used. If applicable, state the seed stock centre and catalogue number. If plant specimens were collected from the field, describe the collection location, date and sampling procedures.

Novel plant genotypes

Describe the methods by which all novel plant genotypes were produced. This includes those generated by transgenic approaches, gene editing, chemical/radiation-based mutagenesis and hybridization. For transgenic lines, describe the transformation method, the number of independent lines analyzed and the generation upon which experiments were performed. For gene-edited lines, describe the editor used, the endogenous sequence targeted for editing, the targeting guide RNA sequence (if applicable) and how the editor was applied.

Authentication

Describe any authentication procedures for each seed stock used or novel genotype generated. Describe any experiments used to assess the effect of a mutation and, where applicable, how potential secondary effects (e.g. second site T-DNA insertions, mosiacism, off-target gene editing) were examined.
