# Supplementary material for: Hepatocellular senescence induces multi-organ senescence and dysfunction via TGFβ
Source: Nat Cell Biol. 2024 Nov 13;26(12):2075–83. doi: 10.1038/s41556-024-01543-3 (PMC11628396; doi:10.1038/s41556-024-01543-3)

Fig.S7g

pSmad2

Unprocessed image used for quantification

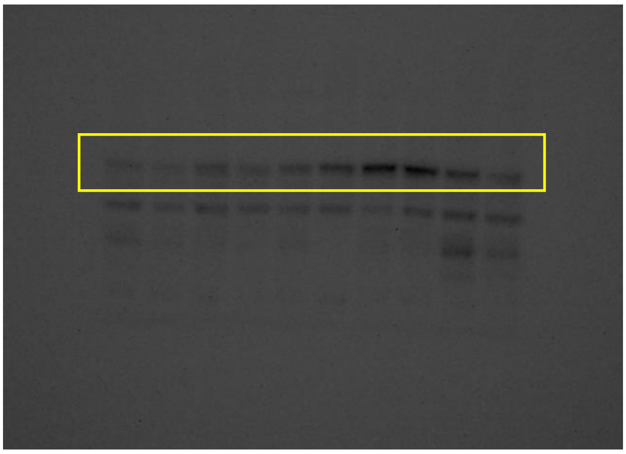

Composite with protein ladder

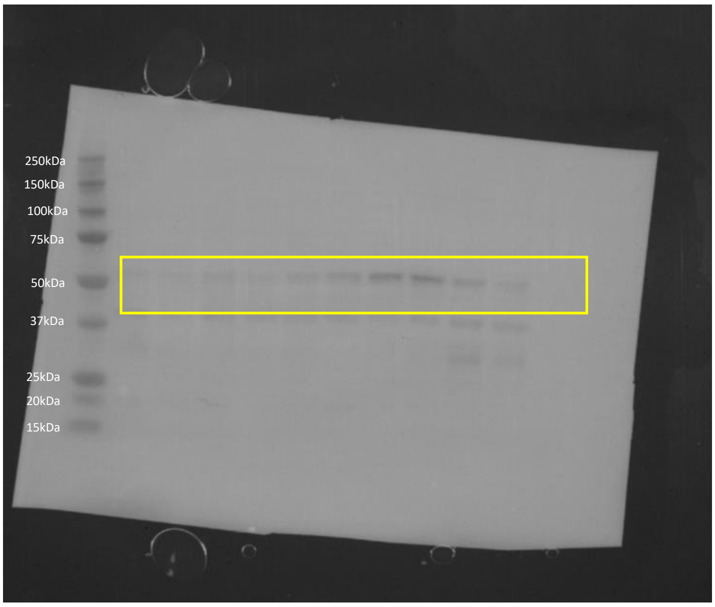

Smad2

Unprocessed image used for quantification

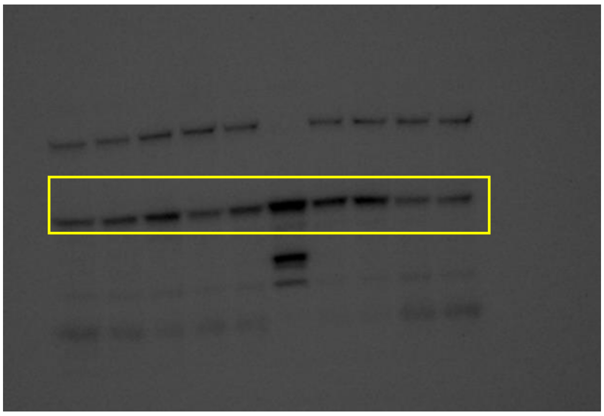

Composite with protein ladder

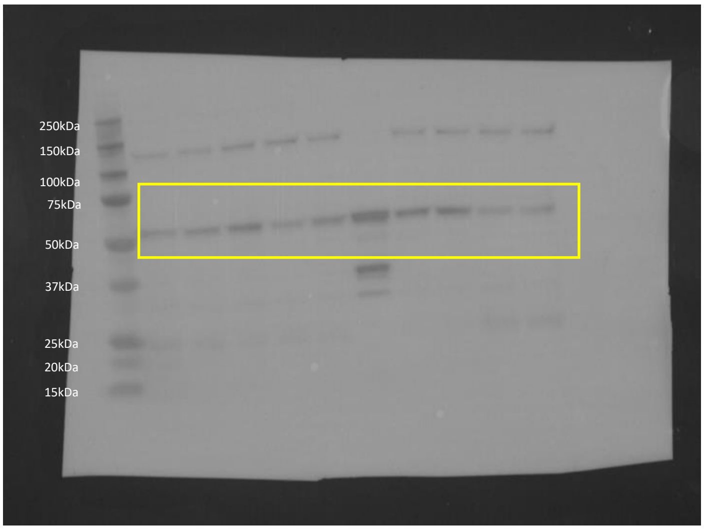

β-actin

Unprocessed image used for quantification

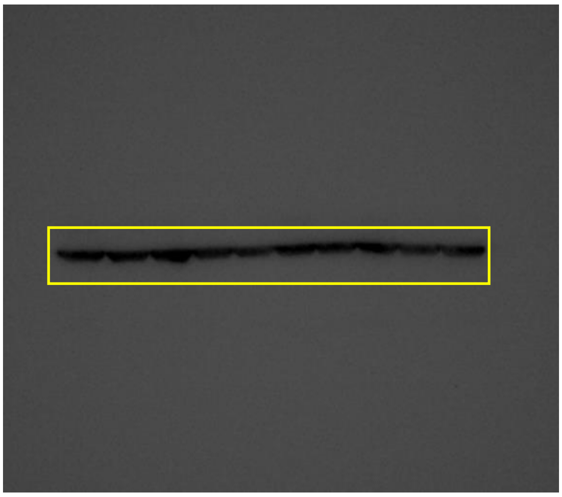

Composite with protein ladder

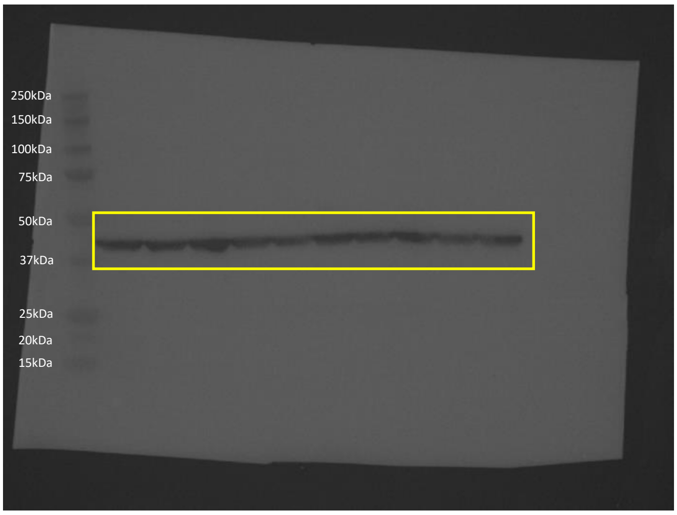

pSmad3

Unprocessed image used for quantification

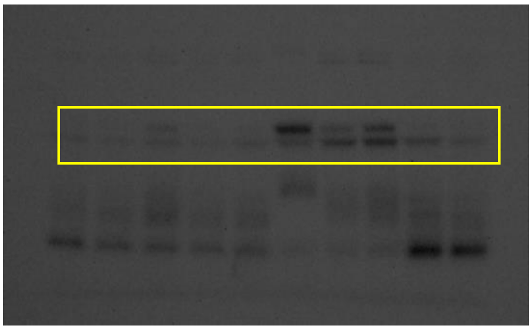

Composite with protein ladder

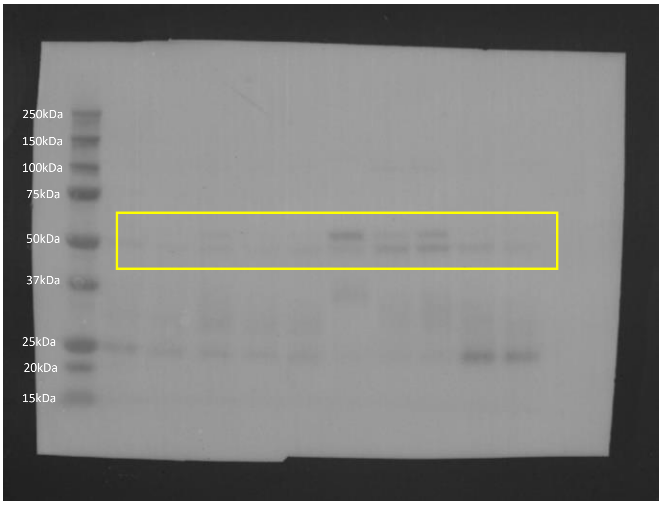

Smad3

Unprocessed image used for quantification

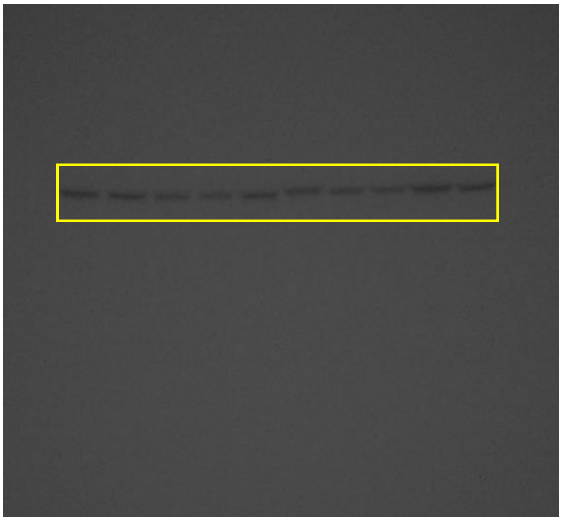

Composite with protein ladder

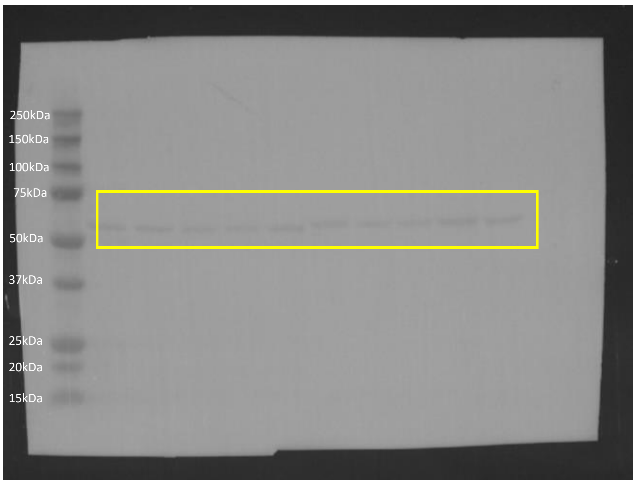

β-actin

Unprocessed image used for quantification

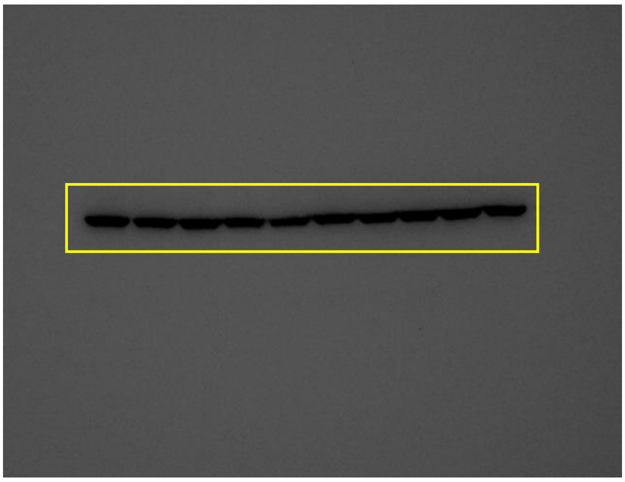

Composite with protein ladder

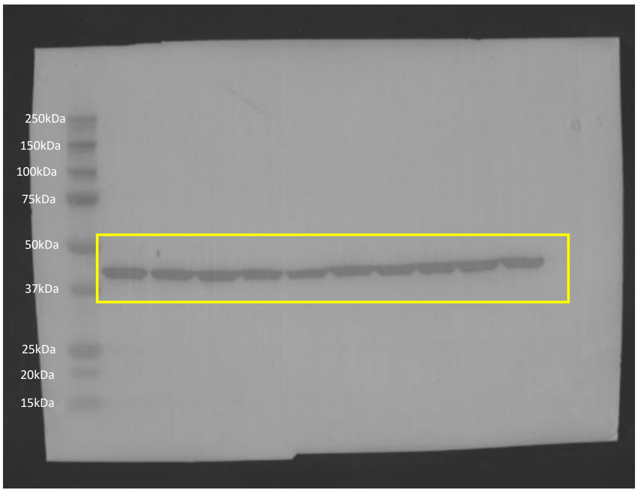

Fig.S8

pSmad2

Unprocessed image used for quantification

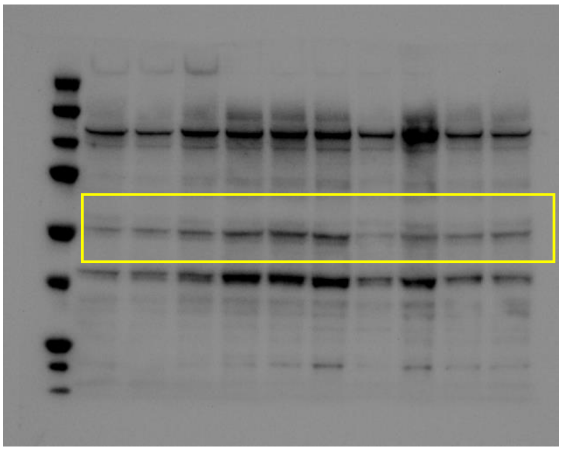

Composite with protein ladder

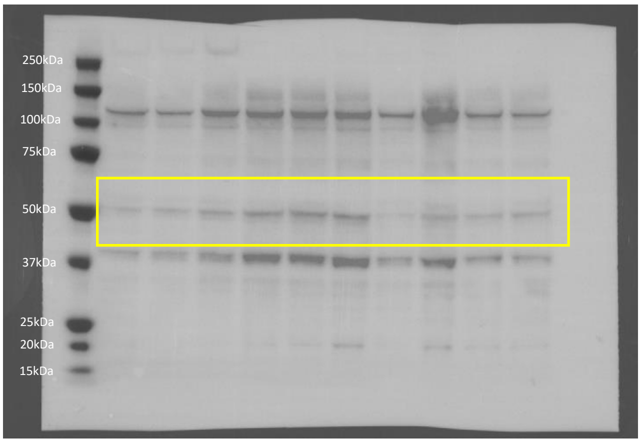

Smad2

Unprocessed image used for quantification

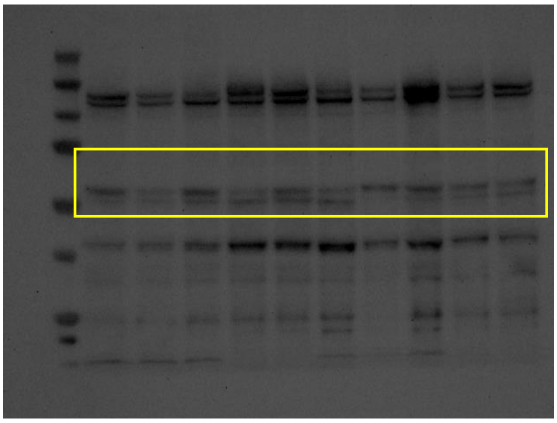

Composite with protein ladder

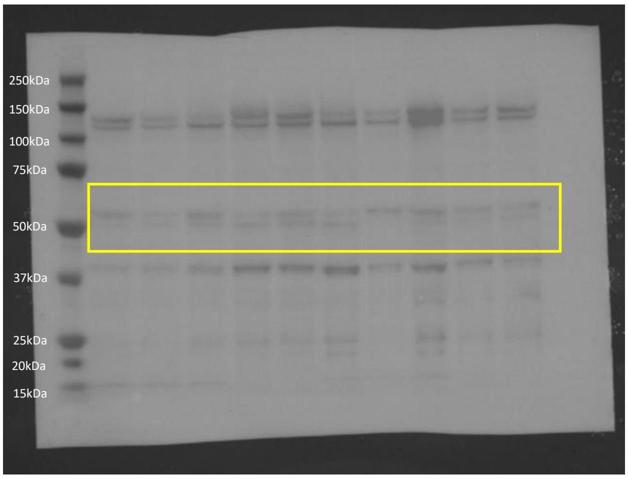

β-actin

Unprocessed image used for quantification

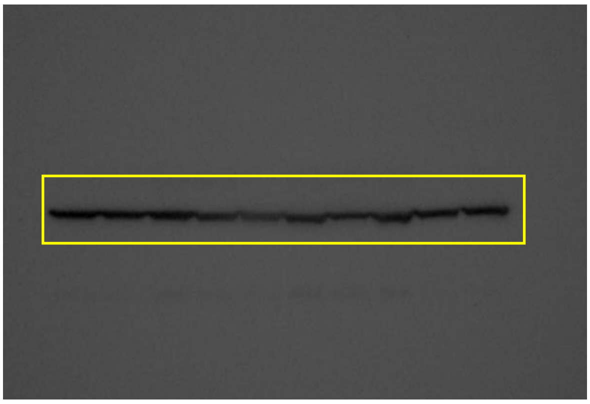

Composite with protein ladder

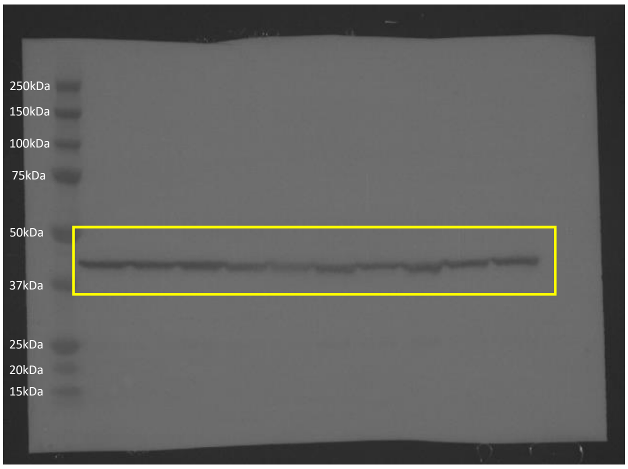

pSmad3

Unprocessed image used for quantification

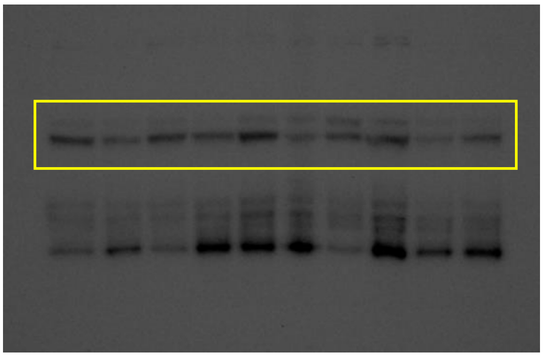

Composite with protein ladder

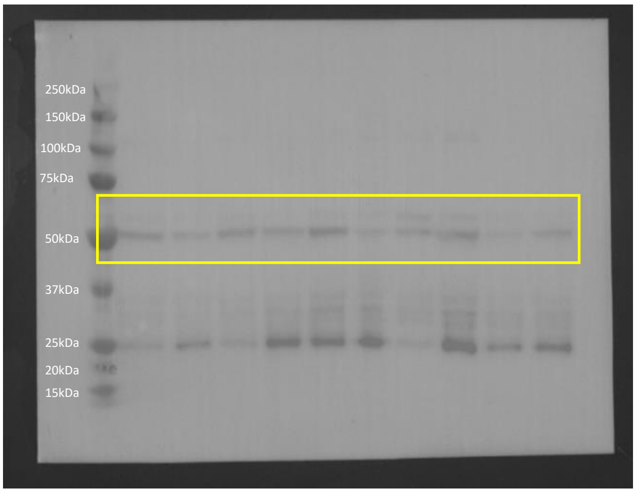

Smad3

Unprocessed image used for quantification

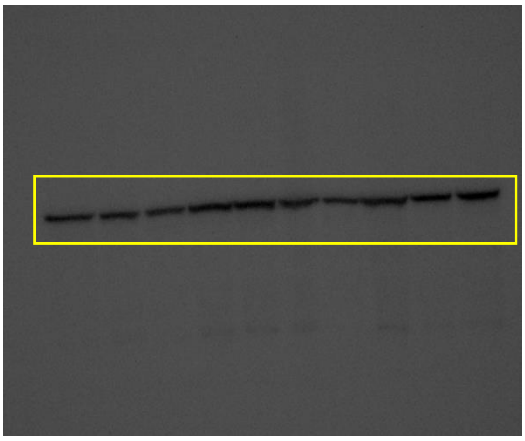

Composite with protein ladder

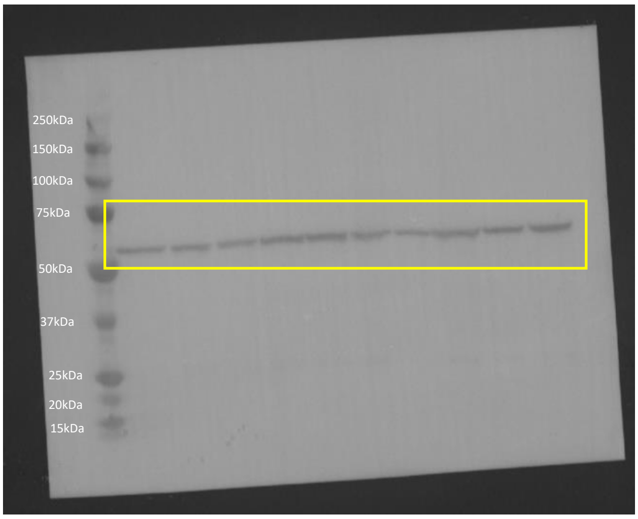

β-actin

Unprocessed image used for quantification

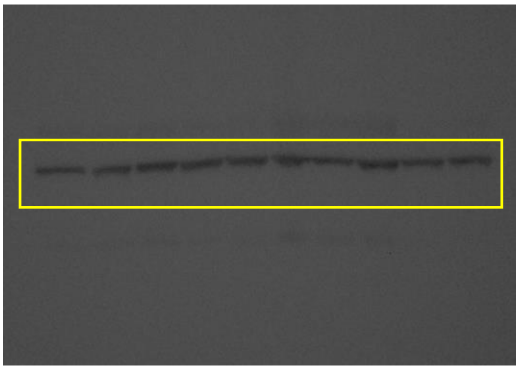

Composite with protein ladder

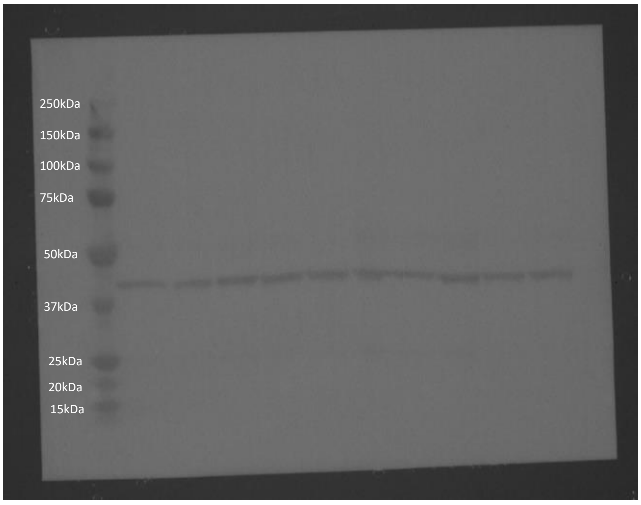

Supplement: Supplementary file 4 — Raw images from western blots. [file 41556_2024_1543_MOESM4_ESM.pdf]
